# Knowledge atlas of antibody-drug conjugates on CiteSpace and clinical trial visualization analysis

**DOI:** 10.3389/fonc.2022.1039882

**Published:** 2023-01-05

**Authors:** Peizhuo Yao, Yinbin Zhang, Shuqun Zhang, Xinyu Wei, Yanbin Liu, Chong Du, Mingyou Hu, Cong Feng, Jia Li, Fang Zhao, Chaofan Li, Zhen Li, Lisha Du

**Affiliations:** ^1^ Department of Oncology, Second Affiliated Hospital of Xi’an Jiaotong University, Xi’an, China; ^2^ School of Chemistry, Xi’an Jiaotong University, Xi’an, China

**Keywords:** antibody-drug conjugates, clinical trials, CiteSpace, Web of Science, research trends

## Abstract

**Objective:**

Antibody-drugs conjugates (ADCs) are novel drugs with highly targeted and tumor-killing abilities and developing rapidly. This study aimed to evaluate drug discovery and clinical trials of and explore the hotspots and frontiers from 2012 to 2022 using bibliometric methods.

**Methods:**

Publications on ADCs were retrieved between 2012 and 2022 from Web of Science (WoS) and analyzed with CiteSpace 6.1.R2 software for the time, region, journals, institutions, etc. Clinical trials were downloaded from clinical trial.org and visualized with Excel software.

**Results:**

A total of 696 publications were obtained and 187 drug trials were retrieved. Since 2012, research on ADCs has increased year by year. Since 2020, ADC-related research has increased dramatically, with the number of relevant annual publications exceeding 100 for the first time. The United States is the most authoritative and superior country and region in the field of ADCs. The University of Texas MD Anderson Cancer Center is the most authoritative institution in this field. Research on ADCs includes two clinical trials and one review, which are the most influential references. Clinical trials of ADCs are currently focused on phase I and phase II. Comprehensive statistics and analysis of the published literature and clinical trials in the field of ADCs, have shown that the most studied drug is brentuximab vedotin (BV), the most popular target is human epidermal growth factor receptor 2 (HER2), and breast cancer may become the main trend and hotspot for ADCs indications in recent years.

**Conclusion:**

Antibody-drug conjugates have become the focus of targeted therapies in the field of oncology. The innovation of technology and combination application strategy will become the main trend and hotspots in the future.

## Introduction

Antibody-drug conjugates (ADCs) have gradually become excellent anti-tumor drugs in recent years. They are formed by a couple of toxic small molecules (payload) with a monoclonal antibody (mAb) through a linker. ADCs deliver highly toxic payloads to specific tumor cells precisely by targeting specific receptors on the surface of tumor cells. This allows the drug concentration of ADCs to be increased only at the tumor site, resulting in an efficient and less toxic antitumor effect. Therefore, ADCs have received much attention from the oncology community in recent years (1). Since 2000, with the breakthrough efficacy of fam-trastuzumab deruxtecan (DS-8201a), ADCs have become a hotspot in the research and development of anti-tumor drugs in the world. Globally, 14 ADCs have been approved by the United States Food and Drug Administration (FDA) for the treatment of various tumor indications, including disitamab vedotin (RC-48) independently developed by RemeGen Co., Ltd. (**Table 1**) (2). With more and more research institutions and pharmaceutical companies entering this field, the clinical application of ADCs has increased. At present, a few kinds of literature systematically evaluate the published related literature. Bibliometrics allows the quantitative analysis of a wide range of literature in a particular field of study using mathematical and statistical methods, revealing many aspects of the field and research trends (3). CiteSpace is a software, which analyzes the co-occurrences and co-citations of a large number of articles in a particular field. It maps the relevant literature into a knowledge map, thus visualizing the trends and research hotspots in this field (4). The National Institutes of Health and the National Library of Medicine jointly opened and operate ClinicalTrials.gov, which collects more comprehensive clinical trials worldwide (5). Using CiteSpace 6.1.R2 software and Excel software, we visualized the concerning literature and clinical trials of ADCs from the WoS platform and ClinicalTrial.gov, respectively. From the results, we sorted out the development of ADCs in the past decade and visualized their hotspots and directions in the future. For future in-depth research and clinical applications of ADCs, this study provides a reference value.

**Table 1:** 14 ADCs on the market.

Year	ADCs	Target	Linker	Payload	Indication
2000	Gemtuzumab Ozogamicin	CD33	Hydrazone linkers	Calicheamicin	CD33-positive acute myeloid leukemia
2011	Brentuximab Vedotin	CD30	Val-cit linker	MORE	Hodgkin lymphoma, Large cell lymphoma, etc.
2013	Ado-trastuzumab Emtansine	HER2	Disulfide linkers	DM1	HER2-positive breast cancer
2017	Inotuzumab Ozogamicin	CD22	Hydrazone linkers	Calicheamicin	Acute B-lymphocyte leukemia
	Gemtuzumab Ozogamicin	CD33	Hydrazone linkers	Calicheamicin	CD33-positive acute myeloid leukemia
2018	Moxetumomab Pasudotox	CD22	Mc-vc-PABC	PEA	Hairy cell leukemia
2019	Polatuzumab vedotin	CD79β	Val-cit linker	MMAE	Diffuse large B-cell lymphoma
	Enfortumab Vedotin-jeff	Nectin-4	Val-cit linker	MMAE	Advanced urothelial carcinoma
	Fam-trastuzumab- Deruxtecan	HER2	Tetrapeptide linker	Dxd	Metastatic HER2-positive breast cancer
2020	Sacituzumab Govitecan-hziy	Trop-2	Hydrolysable CL2A	SN38	Triple negative breast cancer
	Belantamab Mafodotin	BCMA	Maleimidocaproyl	MMAF	Multiple myeloma
	Cetuximab Sarotalocan Sodium	EGFR	—	IRDye 700DX	Head and neck tumor
2021	Loncastuximab Tesirine	CD19	Valine-alanine	PBD	Recurrent or refractory diffuse large B-cell lymphoma
	Disitamab Vedotin	HER2	Val-cit linker	MMAE	Local late-stage HER2 overexpression or metastatic gastric cancer
	Tisotumab Vedotin	TF	Enzyme cleavable linkers	MMAE	Recurrent or metastatic cervical cancer

## Method

### Data collection and retrieval strategy

The literature data used in this study were downloaded from the core collection of the Web of Science retrieval platform, which is widely accepted in bibliometric analyses. The subject retrieval is carried out in the above database by keywords(TS=(“Antibody-Drug Conjugates*” OR “ADCs*”) AND TS=(“cancer*” OR “neoplasm*” OR “tumor*”)), and the retrieval time range is from January 1, 2012, to March 21, 2022, A total of 2510 references were searched. We included articles and reviews, and the letters, editorial materials, conference presentations, meeting abstracts, book reviews, news, and corrections were eliminated. The field of literature was restricted to oncology. Finally, a total of 696 references were eventually retrieved, including 419 articles and 254 reviews.

Clinical trial data come from the website ClinicalTrials.gov, which is the largest and most authoritative clinical trial registration website in the world. All clinical trials must be registered on this website before further development. In this study, the platform ClinicalTrials.gov was selected to collect and analyze the clinical trial data of natural antitumor drugs. This study was searched on the clinical trial registration website ClinicalTrials.gov as “Antibody-Drug Conjugates”. The research type was limited to intervention (clinical trial), and a total of 187 drug trials were in progress before the project was retrieved.

### Data analysis

The 696 documents retrieved in WoS are exported to full records and cited references in plain text format, and then imported into CiteSpace 6.1.R2 software. The parameter settings are adjusted as follows: the period is 2012.01-2022.12, the time slice is 1 year, and the TOP N is set to 50. That is, in the ten years from 2021 to 2022, 50 entries with the highest frequency of occurrence are selected for visualization every year. These documents are visually analyzed for journals, countries, institutions, keywords, and co-cited references, such as cooperative network, clustering, time distribution, salience, etc., and the map is drawn.

We will use Excel to statistically analyze the data of 187 clinical trials related to ADCs collected on the ClinicalTrials.gov platform. Then, we will map from national distribution, drug types, drug targets, cancer classification, clinical stages, etc. to summarize the development trend and research hotspots of ADCs.

## Results

### Distribution by publication time

**Figure 1:**
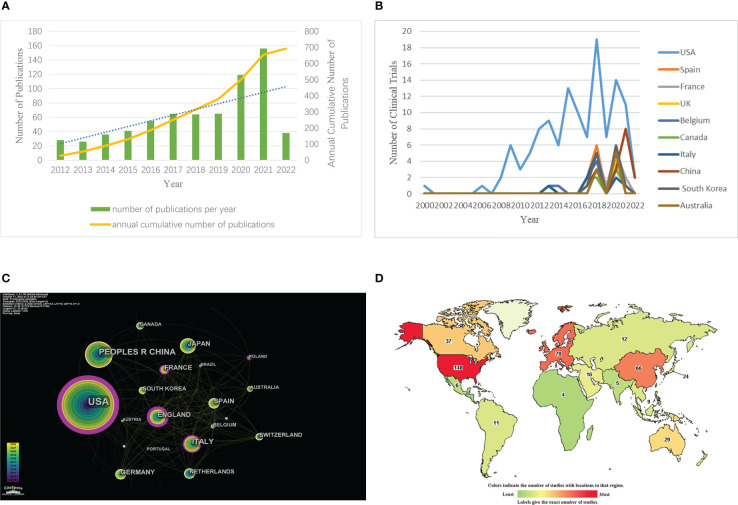
Annual growth trends and geographical distribution of ADCs. **(A)** The number of ADCs-related papers (as of the end of March 2022). **(B)** The co-occurrence map of countries/regions in ADCs research. **(C)** National map of clinical trials in ADCs research. **(D)** National ranking of clinical trials in ADCs research (https://www.clinicaltrials.gov/).

The 696 papers included in the WoS platform were statistically mapped according to the time of publication (**Figure 1**). From **Figure 1**, it can be seen that the number of publications related to ADCs has been increasing year by year during this decade. From 2020 onwards, the annual number of relevant publications on ADCs exceeds 100 for the first time.

### Distribution by country and region

Through CiteSpace, we analyzed the cooperation map of countries or regions with N (number of network nodes) =46 and E (number of connections) =179 (**Figure 1**). According to the analysis, the top 5 countries in the number of documents are the United States, China, Japan, Italy, and the United Kingdom. The top 5 countries in the central ranking are the United States, Britain, Italy, France, and Spain (**Table 2**). A node’s centrality is related to its role in the knowledge network and how it affects other nodes. Nodes with high centrality (>0.1) are usually considered to be turning points or critical points in a domain. Get the global distribution of ADCs on the clinical trial registration website (**Figure 1**). Globally, the regions included on the map are Africa, Central America, East Asia, Europe, the Middle East, North America, North Asia, Pacifica, South America, South Asia, and Southeast Asia. The darker the color is, the more the number of registrations is. The lighter the color is, the less the number of registrations is. The top 10 countries in terms of the number of registered clinical trials were counted by Excel, and then their annual number of registered trials was counted and plotted separately (**Figure 1**). The top 10 countries included the United States, Spain, France, the United Kingdom, Belgium, Canada, Italy, China, South Korea, and Australia. According to the comprehensive analysis, the research output of ADCs is increasing year by year, covering all regions, including the United States, Europe, and Asia. Among them, the United States is far ahead in this field.

**Table 2:** The top 10 countries/regions involved in ADCs.

Countries	Publications	Countries	Centrality
USA	310	USA	0.58
China	91	UK	0.33
Japan	51	Italy	0.20
Italy	48	France	0.12
UK	41	Spain	0.11

### Distribution of institutions

**Figure 2:**
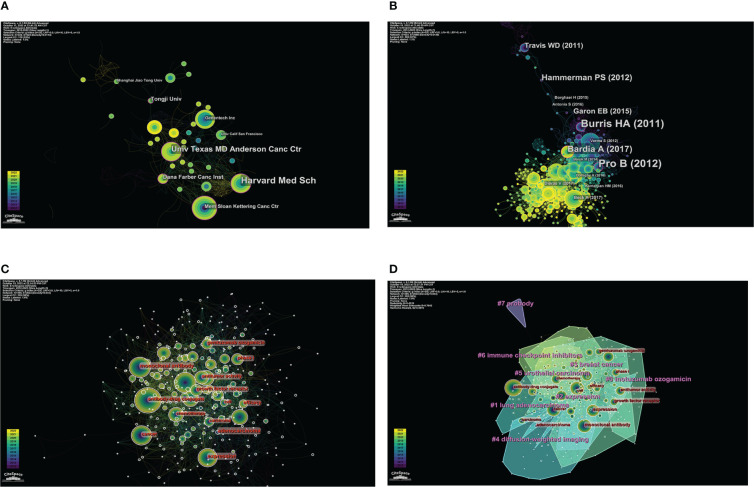
Visual analysis of institutions, co-cited references, and keywords in ADCs research. **(A)** The co-occurrence map of institutions in ADCs research. **(B)** Co-cited network map of references in ADCs research. **(C)** Co-occurrence of keywords related to ADCs by centrality. **(D)** Clustering map of keywords related to ADCs.

CiteSpace analyzed the organization cooperation map (**Figure 2**) with N=332 and E=623. The top five institutions in the number of articles published are MD Anderson Cancer Center, Memorial Sloan-Kettering Cancer Center, Genentech, Harvard Medical School, and Zhejiang University. The top five institutions in the center are Harvard Medical School, Dana-Farber Cancer Institute, MD Anderson Cancer Center, Memorial Sloan-Kettering Cancer Center, and Genentech (**Table 3**). According to the comprehensive number of publications and central analysis, MD Anderson Cancer Center at the University of Texas in the United States can be regarded as an authoritative institution in this field.

**Table 3:** The top 5 institutions involved in ADCs.

Institutions	Publications	Institutions	Centrality
MD Anderson Cancer Center	26	Harvard Medical School	0.14
Memorial Sloan-Kettering Cancer Center	19	Dana-Farber Cancer Institute	0.11
Genentech	19	MD Anderson Cancer Center	0.10
Harvard Medical School	17	Memorial Sloan-Kettering Cancer Center	0.10
Zhejiang University	15	Genentech	0.07

### Analysis of cited references

CiteSpace analysis obtained a network diagram of co-cited references with N=633 and E=2980 (**Figure 2**). The frequency and centrality of citations were used as parameters to statistically rank the top 5 studies, and a total of 12 key node papers were identified. However, since lung adenocarcinoma has the same abbreviation (ADC) as antibody-drug conjugates, further screening of these articles was required. Finally, 10 co-cited references related to antibody-drug conjugates were sifted, and two articles were excluded (6, 7) (**Table 4**). Among these, the articles published by Alain Beck et al. (8) and Shanu Modi et al. (9) were ranked first in terms of the number of citations (citation counts=77). The review by Alain Beck et al. summarized the development of three generations of ADCs and presented the challenges faced by next-generation ADCs. Shanu Modi et al. conducted a phase II clinical trial of DS-8201a, which confirmed its efficacy in HER2-positive metastatic breast cancer with antitumor activity (NCT03248492). Equally important, the phase II multicenter clinical trial of BV for relapsed or refractory systemic interstitial large cell lymphoma (ALCL) by Barbara Pro et al. (10) in 2012 (NCT00866047) had the highest intermediary centrality (centrality=0.21), suggesting that this article plays an excellent pivotal role in the structure of the research literature on ADCs.

**Table 4:** The top 5 co-cited references involved in ADCs.

Publication Year	Author	Citation Counts	Reference	Publication Year	Author	Centrality	Reference
2017/2019	Alain Beck, etc./Shanu Modi, etc.	77	(8, 9)	2012	Barbara Pro, etc.	0.21	(10)
2016	Yusuke Ogitani, etc.	53	(11)	2011	Howard A. Burris III, etc.	0.20	(12)
2019	Gunter von Minckwitz,etc.	49	(13)	2017	Aditya Bardia, etc.	0.17	(14)
2012	Sunil Verma, ect.	46	(15)	2015	Edward B. Garon, ect.	0.14	(16)
2019	Aditya Bardia,etc.	45	(17)				

### Analysis of keywords

Keywords are a high generalization of the content and a reflection of the theme of the article. Keywords with high frequency can usually reflect the research hotspots and the main directions in this field (18). The keyword co-occurrence map was constructed with CiteSpace (**Figure 2**, N=399, E=2624). According to the frequency of occurrence, the keywords “expression”, “cancer”, “monoclonal antibody”, “open-label”, “chemotherapy”, “breast cancer”, “treatment”, “antitumor activity”, “brentuximab vedotin”, “phase II trial”, “trastuzumab emtansine”, “antibody-drug conjugates”, “ HER2” and other keywords were obtained. According to the central order, the keywords “expression”, “monoclonal antibody”, “open-label”, “chemotherapy”, “cancer”, “antitumor activity”, “efficacy”, “phase I”, “phase II trial”, “growth factor receptor” and “adenocarcinoma” were obtained. By using the log-likelihood algorithm, the keywords of the literature are extracted for clustering and naming using CiteSpace. Finally, seven clusters are formed (**Figure 2**). The clustering result is good with Q=0.4319 and S=0.7642. The Q value is greater than 0.3, and the S value is greater than 0.5, which indicates that the clustering results are reasonable. It suggests that the research on ADCs is a hotspot in drugs, therapeutic targets, types of cancer treatment, clinical stages of drugs, and other aspects.

### Analysis of ADCs

**Figure 3:**
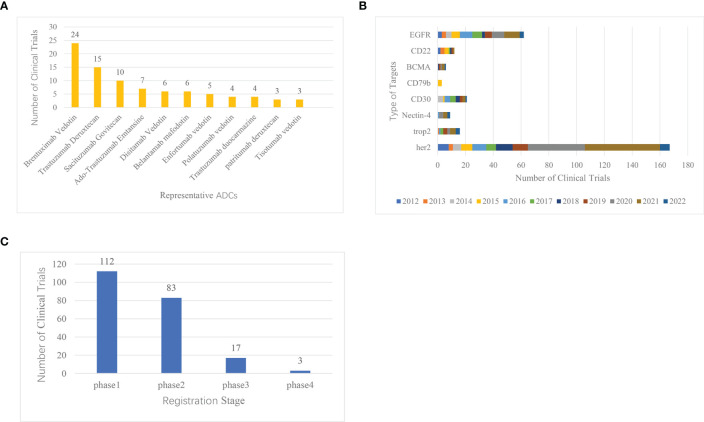
Clinical registration trials of ADCs ranked by representative drugs, common target, and clinical stages. **(A)** Ranking of clinical trials of representative ADCs research. **(B)** Histogram of common targets of ADCs. **(C)** Distribution of clinical trial registration stages in ADCs research.

Statistical analysis was conducted on the website Home-ClinicalTrials.gov on the top 11 ADCs clinical trials in clinical registration. Also, for the wide range of data, we searched these 14 marketed ADCs on http://www.chictr.org.cn as well, searching their Chinese names, English names, trade names, and abbreviations. Finally, we find one clinical registration trial on BV and six clinical registration trials on RC-48. The other 12 ADCs were not found for clinical registration trials. The results of these two sites were aggregated and then mapped for analysis (**Figure 3**). The results showed that BV, DS-8201a, sacituzumab govitecan (IMMU-132), and Ado-trastuzumab emtansine (T-DM1) were the most studied drugs in the field, with BV being the most studied drug in clinical trials.

### Analysis of target

In the design of the ADC, goal selection is the starting point of the ADC and the first component that should be considered in the system (19). On the Home-ClinicalTrials.gov website, we analyzed the common targets for ADCs and ranked the top 8 most studied targets, which are HER2, EGFR, CD30, Trop-2, CD22, Nectin-4, BCMA, and CD79 (**Figure 3**). Over the past decade, the number of clinical trial registrations has increased significantly in 2020, and it is trending upward year by year. One of the most studied ADC targets is HER2, which has 167 clinical trials.

### Analysis of disease

**Figure 4:**
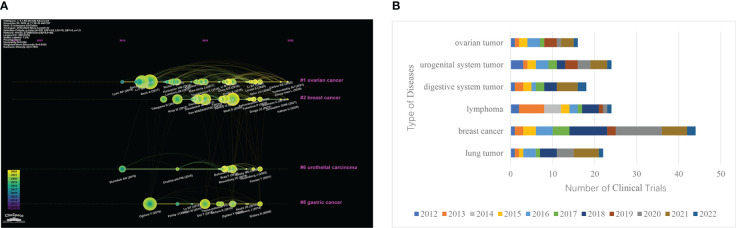
Major clinical indications for ADCs. **(A)** Clustering timeline of references related to ADCs research. **(B)** Bar chart of the clinical trials and disease type ranking of ADCs.

CiteSpace was used to conduct timeline graph cluster analysis on the above co-cited references. And then screening and induction were carried out according to the types of diseases (**Figure 4**). Four clusters were obtained, that is, the four cancer types with the largest number of ADCs. The smaller the number of cluster labels (#), the larger the scale of the cluster. The clustering results were #1 ovarian cancer, #2 breast cancer, #6 urothelial carcinoma, and #8 gastric cancer. The clinical trials of ADCs of common cancer species were counted on the clinical trial registration website Home-ClinicalTrials.gov. And the bar chart statistical analysis was carried out with Excel (**Figure 4**). The top six cancer species were breast tumor, lymphoma, urogenital system tumor, lung tumor, digestive system tumor, and ovarian tumor. Comprehensive analysis shows that the most studied diseases in the field of ADCs are breast cancer, lymphoma, uroepithelial cancer, gastric cancer, and ovarian cancer. Among them, ADCs were the first to reach a peak in the field of lymphoma as early as 2013. Three years later, ovarian cancer and gastric cancer also had breakthroughs in ADCs. In 2018, a large number of clinical trials began to be devoted to the exploration of breast cancer, and it peaked in 2020, which has also shown a gradual upward trend in recent years.

### Analysis of clinical trial stage

According to the results of CiteSpace keyword analysis (**Figure 2**) and the distribution results of ADCs clinical trials registration stage (**Figure 3**), the result shows that there are 112 phase I clinical trials, 83 phase II clinical trials, 17 phase III clinical trials, and 3 phase IV clinical trials. The distribution of the number of phases shows that more than 100 ADCs in clinical trials worldwide have already entered phase III and IV, and have been successfully marketed. With the successful launch of these drugs, more and more ADCs have entered clinical trials, and most of them are still in Phase I and II, suggesting that ADCs are about to reach their peak in the future.

## Discussion

### General information

In this study, we used CiteSpace to conduct a systematic search and analysis of ADCs literature within the WoS core database from 2012-2022. We then used Excel to count the registered clinical trials on ADCs in Home-ClinicalTrials.gov. In total, 696 papers were included and 187 clinical trials were registered after excluding papers that did not meet the screening criteria. In order to provide reference and help for the application of ADCs in targeted cancer therapy, we analyzed the current research status, development trend, and future hotspots of ADCs worldwide.

From the results, there has been a significant increase in papers relating to ADCs over the past few years, indicating that this topic has received much attention (**Figure 1**). In addition, a substantial increase in the number of papers in 2020 could be attributed to the successive launches of sacituzumab govitecan-hziy, belantamab mafodotin, and cetuximab sarotalocan sodium since 2020 and the start of many new ADCs entering clinical trials in various countries, which have boosted research related to the field of ADCs. Global sales of currently marketed ADCs rose significantly from 2020 onwards, and are forecast to exceed US$16.4 billion in 2026 (20). Clearly, ADCs will continue to attract attention over the coming years.

The distribution of ADCs by country and institution is also distinctive. As seen in **Figures 1B**, and **Table 1**, the United States is not only the country with the highest number of published literature (n=310) but also leads in the number of clinical trials (n=148). Moreover, the literature published in the United States is also the most influential in this field (centrality=0.58). It can also be seen from **Figure 2** and **Table 3** that of the top 5 institutions, 5 of the top 6 are United States research institutions. The Chinese research institution of Zhejiang University ranks 5th in terms of the number of publications and is the most published institution in China in this field. Overall, the United States is the first and most influential country in the field of ADCs and has the core research value that puts it far ahead of other countries. China has the second-highest number of published papers (n=91), but the impact of relevant papers is not high. In terms of drug clinical trial research and development, China started to increase significantly in 2020, following the United States, indicating that China has started to focus on the development of ADCs and clinical research in recent years. Overall, research on ADCs is concentrated in a small number of developed countries, mainly in the United States, which may be related to the high funding requirement of ADCs, or the medical technology in developed countries and research collaboration between them.

### Knowledge base

Co-citation is a technique used in research to gauge how relevant two papers are to one another. The knowledge base is a list of references that have been used together (21). In this study, there are 10 papers related to the field of ADC, which are cited most frequently (**Table 4**). Nine clinical trials and one review are included in these journals.

Alain Beck et al. (8)’s high-quality review on strategies and challenges for next-generation ADCs published in 2017 was the most cited (Citation Counts=72). It summarizes the three generations of ADCs and suggests directions for the third-generation ADCs. The other 9 clinical trials outline the progression and breakthroughs of ADCs. The first ADC Mylotarg (gemtuzumab ozogamicin) entered the market in 2000, which represented the successful development of the first generation of ADCs. It used a humanized mAb of IgG4 isotype, coupled to a potent cytotoxic plus ozogamicin *via* an acid-unstable linker. However, the drug was withdrawn from the market in 2010 due to unstable linkers and the easy shedding of toxic drugs (22). In the decade since then, scientists have continued to explore the limitations of the first-generation ADCs development technology, but still without breakthroughs. It was not until after 2011 that the successful clinical trials and successive launches of BV and T-DM1 marked the second stage of the development of ADCs. The second-generation ADCs have improved the design strategy of the connector and optimized the mAb as well as the payload, resulting in significantly improved clinical efficacy and safety of ADCs (23). In 2012, one year after BV was launched, Barbara Pro et al. (10) conducted a phase II multicenter clinical trial on its efficacy and safety in patients with relapsed or refractory systemic ALCL (NCT00866047). This article had the highest centrality (centrality = 0.21), suggesting that the results of this trial are leading the way for future studies in the field of ADCs. The higher remission rate of BV in relapsed or refractory systemic ALCL also provides a reference value for first-line treatment down the road. Notably, ADCs have been of great interest in the field of breast cancer. A single-arm phase II trial of T-DM1 (TDM4258g) conducted by Howard A. Burris III et al. (12) in 2011 opened the way for drug development in the breast cancer field (NCT00509769). This trial validated the safety and efficacy of T-DM1 in patients with HER2-positive metastatic breast cancer (MBC) previously treated with trastuzumab. Two years later, Sunil Verma et al. (15) followed up with a trial (NCT00829166) using T-DM1 in HER2-positive advanced breast cancer patients previously treated with trastuzumab and paclitaxel, and they found that T-DM1 significantly prolonged progression-free (PFS) and overall survival (OS) and was less toxic than lapatinib plus capecitabine. This trial led to the approval of T-DM1 for marketing in 2013. In 2019, G. von Minckwitz et al. (13) again turned their attention to the adjuvant efficacy of T-DM1, they found that adjuvant T-DM1 treatment reduced the risk of recurrence and death in HER2-positive early-stage breast cancer patients with residual invasive breast cancer (NCT01772472). The tremendous success of T-DM1 has prompted an increasing number of scientists and developers to focus on the field of breast cancer. In 2016, DS-8201a was proposed by Yusuke Ogitani et al. (11) as a new antitumor drug candidate that can act on HER2 targets in breast cancer. The authors showed that DS-8201a exhibited potent antitumor activity in T-DM1-insensitive HER2-positive cancers and low HER2-expressing cancers through *in vivo* and *in vitro* assays. This finding initiated scientists to explore the third-generation ADCs DS-8201a. 3 years later, a phase II clinical study on DS-8201a also confirmed its potent killing activity against HER2-positive metastatic breast cancer (NCT03248492) (9). Compared to second-generation T-DM1, third-generation DS-8201a uses a cleavable linker to attach the antibody to the cytotoxic DNA topoisomerase inhibitor (DXd), as well as a targeted coupling technique using cysteine residues. These contribute to its greater lethality and less toxic side effects. In 2021, the DESTINY-Breast 03 phase III trial thoroughly demonstrated the high clinical value of DS-8201a compared to T-DM1 in HER2-positive MBC patients, with statistically significant improvement in PFS (NCT03529110) (24). The successful launch of T-DM1 and DS-8201a has culminated in the development of ADCs in HER2 targets, which has inspired developers to explore other target areas. In 2017, the results of a clinical study on IMMU-132 targeting Trop-2 for triple-negative breast cancer showed good tolerability (NCT01631552) (14). Two years later, A. Bardia et al. (17) also found that IMMU-132 was associated with durable objective responses in heavily pretreated patients with metastatic triple-negative breast cancer (NCT01631552). Since then, the development of Trop-2 targets has proceeded in full swing.

Overall, these 10 highly co-cited papers show the current exploration and development of ADCs, and the analysis of co-cited references provides a deeper comprehension of the architecture of knowledge relevant to ADCs.

### Structure of ADCs and optimization strategy

ADCs consist of three components: antibodies, cytotoxic payload, and linkers. In recent years, the updated iterations of the three generations of ADCs are closely related to the improvement of their three major components and the appearance of new coupling methods. However, the current ADCs still have certain limitations that require further optimization strategies for improvement.

#### Target and antibody

To deliver the cytotoxic payload to cancer cells, ADC targets must be internalized. Therefore, In the design of ADCs, target selection is the starting point of ADCs and the first element that should be considered in the system (19). The selection of ADC targets must meet certain requirements. (1) The target should be abundantly or preferentially expressed on cancer cells and minimally expressed on healthy tissue. (2) The target should also have minimal cancer cell secretion in the circulation, thereby maximizing antibody binding to the target cells (19). (3) The target antigen should be internalized into the cell immediately after binding to the ADC, resulting in a toxic effect on the payload (25). In the last decade, HER2 has been the most studied target in the ADC field and is gradually maturing (**Figure 3**). Human epidermal growth factor receptor 2 (HER2) is a tumor-associated antigen, which is overexpressed in tumor tissues by more than 100 times compared to the equivalent normal tissues (26). Mutations in this antigen are risk factors for carcinogenesis and are expressed in a variety of tumors, such as HER2-positive breast cancer (27), gastric cancer (28), metastatic colorectal cancer (29), and lung cancer (30). Currently, ADCs targeting HER2 for breast cancer include T-DM1, DS-8201a, and trastuzumab duocarmazine (SYD985). RC48, developed by Remegen Ltd, is the first ADC to submit a new drug marketing application in China, which combines an anti-HER2 monoclonal antibody with monomethyl auristatin E (MMAE) *via* a cleavable linker (31). In addition to HER2, several ADCs targeting other targets have been marketed. For example, cetuximab sarotaxol sodium, which targets EGFR, and ADCs targeting related CD targets for the treatment of hematologic and lymphoma diseases, have demonstrated better efficacy in the clinic. However, ADC targets still need to be optimized to reduce toxic effects. In addition to the three requirements for targets mentioned above, the exploration of next-generation ADCs needs to consider the homogeneity of target antigen expression in target-positive patients, as well as antigen shedding and secretion (32, 33). These may lead to reduced ADC binding to the target, which may cause an elevated risk of toxicity. In addition to the optimization of existing targets, the exploration of novel targets is essential for the expansion of the ADC field. Currently, there are novel targets in clinical phase II- III trials, such as folate receptors, tissue factor, LIV-1, CEACAM, CA6, and Nectin-4.

Antibodies are selected based on the identification of molecular targets for which it has the greatest intimacy and selectivity. To avoid inappropriate target-killing effects, it should identify overexpressed targets only at the tumor site (34). Early ADCs used highly immunogenic murine antibodies and human anti-mouse antibodies (HAMA), the latter of which, although an improvement over the former, still had the problem of leading to reduced efficiency and triggering human anti-chimeric reactions (35, 36). Today, the new generation of ADCs is more likely to use chimeric, humanized, or fully human antibodies to solve some of the previous problems. The currently marketed BV uses a chimeric antibody, which consists of a linkage with a human constant fragment (Fc) and a mouse antigen-binding fragment (Fab), as Fc can mediate the interaction with the immune system cells, resulting in reduced immunogenicity. However, it does not prevent the production of human anti-chimeric antibodies (HACA), a risk that is reduced by the humanized antibody trastuzumab used in T-DM1. It consists only of the complementary determining region of Fab transplanted into the human Fab region. To prevent the development of an immune response, a fully human antibody anti-nectin-4 mAb was used for the composition of enfortumab vedotin. However, these antibodies used in modern ADCs can only minimize problems such as acute hypersensitivity reactions or the production of neutralizing drug-resistant antibodies, but these problems have still not been fundamentally solved.

#### Linker

As one of the most difficult aspects of developing ADCs, the selection of linkers is critical, as it promotes the biological coupling reaction. Linkers can prevent the premature release of drugs, improve the release of active drugs at the target and promote the stability of the production process (37). At present, ADC linkers that have been listed and are in clinical trials can be mainly divided into two categories, namely, cleavable linkers and non-cleavable linkers (**Table 1**). Cleavable linkers are also divided into hydrazone linkers, disulfide linkers, and enzyme cleavable linkers. Hydrazone linkers are often coupled with doxorubicin, auristatin E, and calicheamicin. gemtuzumab ozogamicin and inotuzumab ozogamicin, which have been approved by FDA, use hydrazone linkers (38). However, the instability of hydrazone linkers led to the withdrawal of Pfizer’s Mylotarg in 2010. It was reintroduced in 2017 after its improvement in hepatotoxicity. Disulfide linkers are often coupled with maytansine and thiol-containing maytansinoid (39). Lorvotuzumab mertansine, coltuximab ravtansine, and anetumab ravtansine, which are in the stage of clinical research, use disulfide linkers. Enzyme cleavable linkers have better plasma stability than the first two kinds of linkers, which can be divided into peptide linkers and β-Glucuronidases linkers. Dipeptide linkers Val-Citrulline-PABC (Val-CIT-PABC) and Val-Ala-PABC (Val-Ala-PABC) in peptide linkers are widely used in ADCs, among which BV, which has been listed, is the most representative. β-Glucuronidases linkers have hydrophilic advantages, which can be coupled with highly hydrophobic drug toxins such as CBI groove binder, psymberin, camptothecin, adriamycin analogues, etc., to prepare ADCs (40). In contrast, the non-cleavable linker has better stability in plasma. For the payload to be released, the entire antibody-linker must be degraded by the lysosome, which usually results in charged amino acids remaining. By doing so, it impairs the action of the protein or the permeability of cells (41). At present, the most commonly used non-cleavable linker is a succinimide-thioether bond, which is usually coupled with maytansinoid derivatives DM1 and auristatin (17). The most representative ADCs are T-DM1 and depatuxizumab mafodotin, the former is coupled with DM1, and the latter is prepared by coupling with monomethyl auristatin F (MMAF). However, the succinimidyl-4-(N-maleimidomethyl) cyclohexane-1-carboxylate (SMCC) linker used in T-DM1 has a retro-Michael elimination reaction, which leads to reduced efficacy of ADC (42). In response to the problems of previous linkers, scientists have been working this year to develop new linkers and conjugation technology to improve the efficacy of ADCs. New chemical triggers (cBu trigger, silyl ether trigger, TRX trigger, photo-responsive cleavable triggers, and bio-orthogonal cuttable trigger) are already being developed, which improve the selectivity in tumor delivery. Compared to the previous conjugation technology (conjugated in random variable positions), the new site-specific conjugation technology has a well-defined drug-to-antibody ratio (DAR), which reduces ADC heterogeneity and improves the therapeutic window (43). The main site-specific conjugation strategies include the use of engineered cysteine residues, unnatural amino acids, and enzymatic conjugation through glycotransferases and transglutaminases. However, many issues remain to be addressed, including off-target toxicity due to instability, drug resistance in cancer cells, retro-Michael elimination reaction of the maleimide linker, heterogeneity of the DAR, and insufficient linker-payload connectivity. All these issues suggest that the technological update of the linker is closely related to the breakthrough of ADC (44–46).

#### Payload

The payload is critical for ADCs to exert cytotoxic effects, and it must have several characteristics. (1) The IC50 values should be low nanomolar or picomolar, which indicates a high level of cytotoxicity. (2) It should have a clearly defined target and a clear mechanism of action. (3) An attachment site for chemicals should be present (47). In addition, the payload should have sufficient stability in plasma. At the same time, the cells die and release toxins to kill neighboring cells, which requires the toxins to be able to cross the cell membrane and thus exert a bystander effect. The payload in ADCs can be divided into two categories, microtubule-disrupting drugs, and DNA-damaging drugs. Microtubule-disrupting drugs include auristatin and maytansinoid derivatives, and ADCs with auristatin as payload generally adopt MMAE and MMAF. BV, which is currently listed in ADC, uses MMAE as a payload to kill tumor cells (**Table 1**). At the same time, more than half of clinical ADCs in research use this payload (47). DM1 and DM4 are commonly used as maytansinoid derivatives, which are powerful microtubule-disrupting drugs. The payload of T-DM1, which is currently on the market, is DM1, which has become a typical representative of maytansinoid derivatives in ADCs. DNA-damaging drugs include camptothecin, calicheamicin, and anthramycin. The principle of camptothecin and its derivatives is to selectively act on DNA topoisomerase I (Topo I), stabilize DNA Topo I complex, block DNA replication and transcription, and lead to tumor cell apoptosis. SN-38 is the most potential camptothecin derivative. Labetuzumab govitecan (IMMU-130) for the treatment of recurrent or refractory colorectal cancer, and IMMU-132 for the treatment of metastatic triple-negative breast cancer, both use SN-38 as a payload to couple with antibodies. In Calicheamicin, N-acetyl-γ- calicheamicin is widely used as the classic payload of ADCs, and gemtuzumab ozogamicin and inotuzumab ozogamicin approved by FDA currently use it. Pyrrolobenzodiazepine (PBD), one of the anthramycin derivatives, has also been gradually used as a payload of new ADCs. The mechanism of action of PBD is to kill tumor cells by binding and cross-linking to specific targets of cancer cell DNA, and it prevents the proliferation of tumor cells without deforming their DNA helix in the process, thus also avoiding the emergence of drug resistance (48). It is the third emerging payload after auristatin and maytansinoid, and will also become the hotspot direction of ADCs payload at present. In addition to this, RNA polymerase inhibitors (such as amatoxin, spliceostatin C, and thailanstatin A) are also expected to be new payloads (26), and they have the advantage of being able to overcome multidrug resistance (MDR) phenotypes compared to MMAE and maytansinoids (49). There are also relevant studies for nitric oxide (NO) as a payload that have shown good efficacy results (50). Currently, more effective and safe payloads are needed to support the development of new ADCs. Moreover, the problems of finding the best DAR and overcoming multi-drug resistance techniques are also needed to be overcome in the future.

In summary, there are still many limitations in ADC technology development. Among them, the off-target toxicity of ADC is the biggest challenge at present. In addition, the problem of ADC resistance and protein aggregation are also the major obstacles to ADC development (51). These issues will be the main focus of ADC in future research.

### Research hotspots

In recent years, with in-depth research on the mechanism of ADC targeting therapy, ADC technology has been greatly innovated and progressed. At the same time, more and more new drug development companies have started to increase the development process of ADCs. Consequently, ADCs have become more widely applied in clinical settings.

In 2021, Carolina do Pazo et al. (20) forecasted the oncology market for ADCs over the next five years and projected that global sales of marketed ADCs will exceed $16.4 billion by 2026. This report also suggests that DS-8201a will be the highest-selling ADC in 2026 ($6.2 billion in global sales). Moreover, enfortumab vedotin ($3.5 billion in 2026), IMMU-132 ($1.1 billion in 2026), and BV ($1.8 billion in 2026) are also expected to have a large market and will be the popular research topics in the future. Trastuzumab Deruxtecan (DS-8201a) has the highest buzz thanks to its broad-spectrum anticancer effects. Since its development, DS-8201a has expanded from breast cancer to include gastric, lung, and colorectal cancers. While the results of the DESTINY-Breast03 trial have demonstrated the tremendous benefits of DS-8201a over T-DM1 in patients with HER2-positive MBC, its exploration in the breast cancer field has not stopped there. The results of the DESTINY-Breast04 trial announced this year showed that DS-8201a prolonged mPFS and mOS compared to chemotherapy in patients with HER2 low expression breast cancer (NCT03734029) (52). Based on this trial, DS-8201a was approved by the FDA for unresectable or metastatic HER2 low-expression breast cancer in August of this year. This approval officially makes HER2 low-expression breast cancer a new breast cancer classification, thus allowing more patients to benefit from HER2-targeted therapy, especially those with what would otherwise be considered triple-negative breast cancer. In the field of gastric cancer, the results of DESTINY-Gastric01 showed a significantly prolonged OS and a significantly improved ORR in DS-8201a compared to the chemotherapy group (NCT03329690) (28). As a result, DS-8201a was approved by the FDA in January 2021 for patients with locally advanced or metastatic HER2-positive gastric or gastroesophageal junction (GEJ) adenocarcinoma who have received prior trastuzumab treatment. This is the first ADC approved for HER2-positive gastric cancer, and in recent years, DS-8201a has also been a breakthrough in lung cancer. both the DESTINY-Lung01 (NCT03505710) (53) and DESTINY-Lung02 (NCT04644237) trials demonstrated a higher OR for DS-8201a in patients with HER2-positive non-small cell lung cancer (NSCLC). As a result, the FDA accelerated the approval of DS-8201a for unresectable or metastatic HER2-positive NSCLC that had received systemic therapy this past August, making it the first HER2-targeted therapy for lung cancer. the success of DS-8201a in these areas has in turn inspired scientists to explore the field of colorectal cancer. the results of the DESTINY-CRC01 trial in 2021 confirmed its antitumor effect in patients with HER2-positive metastatic colorectal cancer (mCRC) (NCT03384940) (29). In the future, DS-8201a will have a huge market as the most sought-after ADC. Also with broad therapeutic promise is IMMU-132, approved in 2020 for the treatment of triple-negative breast cancer, which is the first ADC approved by the FDA to target Trop-2. In addition to triple-negative breast cancer, IMMU132 has demonstrated excellent clinical efficacy in several solid tumors, including lung cancer (NSCLC and SCLC) (54, 55), uroepithelial cancer (56), colon cancer (57) and ovarian cancer (58). This is attributed to the expression of Trop-2 in several cancer types. Similar to HER2, Trop-2 will be a new hot target for research after HER2.

In addition to the fact that some ADCs have multiple indications in clinical practice, combination therapeutic strategies have become an important research direction in the current clinical treatment of ADCs. A reasonable combination strategy can improve the activity of ADCs and achieve more accurate and higher killing ability. Currently, relevant clinical studies have confirmed the superiority of combination strategies for ADCs. BV’s combination chemotherapy regimen BV + AVD (doxorubicin, vinblastine, and dacarbazine), has been approved in 2018 for the treatment of previously untreated adult patients with stage III or IV classic HL (NCT01657331) (59). BV was also included in the combination chemotherapy regimen BV + CHP (cyclophosphamide, doxorubicin, and prednisone) in 2020 for the first-line treatment of adult patients with CD30-positive systemic mesenchymal large cell lymphoma (sALCL) (60). The combination of Neratinib and T-DM1 in the same lung human tumor xenograft model (PDXS) with ERBB2 amplification and mutation (S310F) has a more significant and lasting effect than that of T-DM1 alone in inducing tumor regression (61). KATE2 clinical trial is the first randomized, double-blind phase II clinical study exploring ADC combined with immunotherapy, (NCT02924883). The results show that the median PFS of T-DM1 combined with the atezolizumab group is 8.5 months, which is significantly longer than that of the T-DM1 plus placebo control group (62). At present, the clinical research on ADCs combined regimen has been in full swing, which indicates that ADCs will provide better curative effects to patients in the future.

## Strengths and limitations

In our research, the bibliometrics software CiteSpace and the statistical software Excel were used to visually and objectively analyze the development trend and research status of antibody-drug conjugates in the past decade. This may provide guidance for clinicians and academics working in this field. Inevitably, this study has some limitations. In the first place, our study does not include a comprehensive review of the literature, we have only included the literature from the WoS core database. CNKI data, as well as PubMed, Scopus, and other important search engines were not included. Similarly, the clinical trial data we included are all from the Home-ClinicalTrials.gov website, and the data from other clinical trial websites are also excluded. Secondly, our study may have ignored some newly published articles of high quality. This is because they were published relatively recently and do not yet have high citation rates. We expect a more comprehensive hotspot analysis to update our study in the future. Finally, although our study data are lacking (publications published in 2022 and data from other clinical trial sites are excluded), this study includes the great bulk of articles published in ADCs between 2012 and 2022, and clinical trial.org is also the largest clinical trial site in the world. Therefore, the new data may have little impact on the final results.

## Conclusion

In general, antibody-drug conjugates have become the focus of targeted therapy in the tumor field in recent years. Breast cancer is the most popular indication in the ADCs research field at present. The development test of ADCs targeting HER2 and Trop-2 targets by various companies is also in full swing. At present, it is in the third-generation research stage of ADCs technology. The specificity and cytotoxicity of the new-generation ADCs are superior to those of the previous-generation products. As the ADC development technology continues to advance, the clinical aspects of the drug are becoming more widely used. Scientists are beginning to explore the efficacy of individual drugs in multiple indications, and combination therapeutic strategy is also becoming a future research direction. However, many challenges remain in the development of ADCs, including the exploration of pharmacokinetic mechanisms, the issue of off-target toxicity, the exploration of new coupling modalities, and the issue of drug resistance. The innovation of ADCs technology and the joint application strategy is still the favored breakthrough points in the field of cancer in the future.

## Data availability statement

The original contributions presented in the study are included in the article/supplementary material. Further inquiries can be directed to the corresponding authors.

## Author contributions

PY is the first author, YZ is the corresponding author and SZ is the co-corresponding author. YZ and SZ contributed to conception and design of the study. CD, MH, CF organized the database. JL, FZ and LD performed the statistical analysis. XW and YL prepare all the figures and tables. PY and CL wrote the first draft of the manuscript. All authors contributed to the article and approved the submitted version.
